# Post-COVID-19 Rehabilitation Improves Mobility and Gait Performance: Evidence from TUG and 10MWT

**DOI:** 10.3390/healthcare13222892

**Published:** 2025-11-13

**Authors:** Ovidiu Cristian Chiriac, Daniela Miricescu, Corina Sporea, Silviu-Marcel Stanciu, Dragos Constantin Lunca, Silviu Constantin Badoiu, Ileana Adela Vacaroiu, Raluca Mititelu, Raluca Grigore, Ana Raluca Mitrea, Sarah Adriana Nica

**Affiliations:** 1 Discipline of Balneophysiokinetotherapy and Recovery, Faculty of Midwifery and Nursing, Carol Davila University of Medicine and Pharmacy, 050474 Bucharest, Romania; ovidiu-cristian.chiriac@drd.umfcd.ro; 2 Central Military Emergency University Hospital “Dr. Carol Davila”, 010825 Bucharest, Romania; 3 Discipline of Biochemistry, Faculty of Dentistry, Carol Davila University of Medicine and Pharmacy, 8 Eroii Sanitari Blvd, 050474 Bucharest, Romania; daniela.miricescu@umfcd.ro; 4 Scientific Research Core, National University Center for Children’s Neurorehabilitation “Robănescu-Pădure”, 44 Dumitru Minca Street, 041408 Bucharest, Romania; 5 Department of Internal Medicine and Gastroenterology, Carol Davila University of Medicine and Pharmacy, Central Military Emergency University Hospital, 050474 Bucharest, Romania; silviu.stanciu@umfcd.ro; 6 Discipline of Pharmacology, Clinical Pharmacology, and Pharmacotherapy, Carol Davila University of Medicine and Pharmacy, 050474 Bucharest, Romania; dragos.lunca@umfcd.ro; 7 Department of Anatomy and Embryology, Faculty of Medicine, Carol Davila University of Medicine and Pharmacy, 8 Eroii Sanitari Blvd, 050474 Bucharest, Romania; silviu.badoiu@umfcd.ro; 8 Department of Nephrology, Faculty of Medicine, Carol Davila University of Medicine and Pharmacy, 020021 Bucharest, Romania; 9 Department of Nuclear Medicine, Carol Davila University of Medicine and Pharmacy, 030147 Bucharest, Romania; raluca.mititelu@umfcd.ro; 10 Clinic of Nuclear Medicine, Central University Emergency Military Hospital “Dr Carol Davila”, 010825 Bucharest, Romania; 11 ENT, Head & Neck Surgery Department, Carol Davila University of Medicine and Pharmacy, Coltea Clinical Hospital, 030171 Bucharest, Romania; raluca.grigore@umfcd.ro; 12 Physical Medicine and Rehabilitation (Medical Recovery Neurology), Carol Davila University of Medicine and 21 Pharmacy, 050474 Bucharest, Romania; ana-raluca.vacaroiu@rez.umfcd.ro; 13 Department of Physical Medicine and Rehabilitation, Carol Davila University of Medicine and Pharmacy, 050474 Bucharest, Romania; sarah.nica@umfcd.ro

**Keywords:** infection, mild COVID-19, moderate COVID-19, post-COVID rehabilitation, functional mobility, Timed Up and Go test (TUG), 10-Meter Walk Test (10MWT), gait speed, physical inactivity, sedentarism

## Abstract

**Background and Objectives**: COVID-19 has been associated with prolonged inactivity and reduced physical performance, even in mild and moderate cases. This study aimed to evaluate changes in functional mobility and gait speed, assessed with the Timed Up and Go (TUG) and 10-Meter Walk Test (10MWT), in patients with mild to moderate post-COVID-19 conditions undergoing a structured rehabilitation program. **Materials and Methods**: A controlled observational study was conducted on 193 patients (115 women, 78 men) who had recovered from mild to moderate COVID-19. Participants were divided into a rehabilitation group (n = 160) and a control group (n = 33) who did not undergo structured physical therapy. Functional performance was assessed with TUG and 10MWT at admission and at one-year follow-up. **Results**: Both tests showed significant improvements following rehabilitation. In the rehabilitation group, the proportion of patients classified as functionally independent increased significantly for both the TUG (Cramér’s V = 0.468, *p* < 0.001) and 10MWT (Cramér’s V = 0.500, *p* < 0.001). The McNemar test confirmed a moderate within-group improvement for 10MWT (*p* = 0.001). Older adults (≥60 years) exhibited functional gains comparable to younger participants. A strong association between final TUG and 10MWT categories (Cramér’s V = 0.40, *p* < 0.001) confirmed the consistency of outcomes. **Conclusions**: Structured rehabilitation significantly improves balance, gait speed, and functional independence in mild-to-moderate post-COVID-19 patients. These findings highlight that rehabilitation should be integrated into the continuum of post-COVID care, as meaningful recovery is achievable even outside severe cases.

## 1. Introduction

Severe acute respiratory syndrome coronavirus-2 (SARS-CoV-2) causes coronavirus disease 2019 (COVID-19) and is primarily transmitted through respiratory droplets and direct contact. The most frequent symptoms in symptomatic individuals include fever, dry cough, and fatigue, while upper respiratory manifestations may also present as sore throat, headache, and myalgia [1,2]. Although COVID-19 primarily targets the respiratory system, leading to persistent pulmonary dysfunction, reduced diffusion capacity, and decreased exercise tolerance [3,4,5,6], it also exerts significant cardiovascular effects. Multiple studies have reported myocardial injury, arrhythmias, myocarditis, endothelial dysfunction, and an increased risk of thromboembolic events associated with SARS-CoV-2 infection [7,8,9,10]. The pathophysiology appears multifactorial, involving viral invasion of myocardial tissue, microvascular inflammation, and systemic hypercoagulability. Importantly, individuals with pre-existing cardiopulmonary conditions are at higher risk of severe disease and prolonged recovery, which further underscores the need for targeted rehabilitation to restore both respiratory and functional capacity [11,12].

In line with internationally recognized guidelines, the present study applies standard definitions of COVID-19 severity. The mild category includes patients who presented with general symptoms such as fever, fatigue, cough, or anosmia, without evidence of shortness of breath, dyspnea, or abnormal findings on chest imaging. In contrast, the moderate group refers to patients with clinical or paraclinical signs of respiratory involvement—such as dyspnea, oxygen saturation below 94%, or the need for supportive medical care. This classification is consistent with the criteria outlined by the National Institutes of Health (NIH) and the World Health Organization (WHO) [13,14,15].

The COVID-19 pandemic and its associated social restrictions have led to widespread reductions in physical activity. This sharp decline in mobility and exercise capacity can exacerbate underlying conditions and contribute to new functional deficits. The World Health Organization therefore recommends that adults (aged 18–64) engage in 150 min of moderate-intensity aerobic activity or 75 min of vigorous-intensity activity per week, or an equivalent combination, to maintain health [16,17]. Evidence indicates that individuals who maintain regular activity are less likely to be hospitalized, require intensive care, or die compared to those who remain sedentary. Physical activity provides protective effects via modulation of inflammation, enhancement of immune response, and reduction in cardiovascular risk [18]. The psychological burden of quarantine and isolation further compounds the risk: studies have documented elevated rates of stress, depression, sleep disturbance, and irritability during periods of restricted mobility, particularly among older adults [19]. Recent studies confirm that post-COVID rehabilitation should address both physical and psychological aspects of recovery. For example, our group has shown that structured physical rehabilitation programs improve not only functional outcomes, but also symptoms of anxiety and depression in patients recovering from COVID-19 [20,21,22].

Functional mobility and gait speed represent critical indicators of health and independence, especially in populations recovering from systemic illnesses. The Timed Up and Go (TUG) test, originally introduced by Podsiadlo and Richardson in 1991, is a widely used tool for assessing dynamic balance and basic functional mobility in elderly populations [23]. Subsequent studies have applied TUG across various clinical settings, including frailty screening and rehabilitation contexts, to identify mobility limitations and assess fall risk through quantitative analysis [24,25,26,27,28].

Meanwhile, the 10-Meter Walk Test (10MWT) is a reliable and validated method of measuring gait speed—a key marker of physical function and predictor of health outcomes. It is often used alongside longer walk tests (e.g., 2 min or 6 min walk tests) to capture deficits in ambulatory capacity [29]. The test has been used across oncological [29], geriatric [30,31], and digital health/technology-based application [32] contexts. In post-COVID patients, emerging evidence demonstrates that both balance and walking speed are affected even in mild cases [33,34,35].

Respiratory and functional impairments have been documented in prospective cohorts of COVID-19 survivors. Reduced lung function and decreased 6-Minute Walk Test performance have been reported up to several months post-infection [36] while persistent fatigue, muscle weakness, and mitochondrial dysfunction have been identified even in younger survivors [37]. Comprehensive pulmonary rehabilitation has demonstrated benefits in patients with severe COVID-19, improving exercise tolerance and quality of life [38,39]. Additionally, Mooren et al. (2023) reported that post-COVID-19 syndrome patients benefitted from aerobic endurance training—both continuous and interval modalities—showing improved exercise capacity, reduced fatigue, and enhanced psychological well-being, further reinforcing the effectiveness of structured rehabilitation even in less severe cases [40]. However, most published data target individuals with severe illness or prolonged hospitalization, leaving those with mild to moderate disease underrepresented in the literature. Recent studies also highlight that functional and respiratory sequelae persist even among young adults recovering from mild disease, underscoring the necessity of structured rehabilitation interventions [41].

Previous research has further emphasized the importance of functional tests in evaluating rehabilitation outcomes. For example, the Six-Minute Walk Test (6MWT) has been proven to be a robust tool in assessing the efficacy of rehabilitation programs in post-COVID populations [36,42,43,44,45]. These findings support the value of combining short-distance tests such as the TUG and 10MWT with longer assessments like the 6MWT to capture the multidimensional aspects of recovery.

Given these considerations, the primary aim of the present study was to evaluate changes in mobility, measured through the TUG test, and gait speed, assessed by the 10MWT, in patients with mild to moderate post-COVID-19 conditions undergoing a structured rehabilitation program. By focusing on this underexplored subgroup, we seek to provide evidence for the importance of rehabilitation strategies not only in severe COVID-19 survivors, but also in those recovering from less critical forms of the disease.

## 2. Materials and Methods

### 2.1. Study Design and Patients

This retrospective observational study with a control group included 193 patients (115 women, 78 men) with mild to moderate post-COVID-19 conditions who were evaluated at the “Dr. Carol Davila” Military Emergency Hospital, Bucharest. The study group consisted of 160 patients (97 women and 63 men) who participated in the structured physical rehabilitation program. The control group comprised 33 patients (18 women and 15 men) with comparable demographic and clinical characteristics who did not undergo rehabilitation but received standard medical follow-up during the same period.

Besides healthy patients, some participants in the study had dyslipidemia and arterial hypertension (37.5%), while others had type 2 diabetes mellitus (12%), and all were receiving specific treatment and able to participate in the recovery program. Patients with chronic circulatory failure, chronic respiratory failure, or chronic musculoskeletal diseases were excluded.

All participants provided informed consent prior to inclusion. The study protocol was approved by the hospital’s Ethics Committee (approval no. 694/28.03.2024).

COVID-19 severity was classified according to internationally recognized guidelines: mild cases included patients with symptoms such as fever, fatigue, cough, or anosmia without dyspnea or abnormal chest imaging, whereas moderate cases presented with signs of respiratory involvement (e.g., dyspnea, oxygen saturation <94%) or required supportive care.

### 2.2. Rehabilitation Program

The rehabilitation protocol combined in-hospital sessions with a home-based continuation phase. During hospitalization, patients participated in supervised low-intensity physical exercises targeting both upper and lower limb muscle tone, respiratory training, and trunk mobility exercises to facilitate verticalization and ambulation. Progressive gait training was performed under the supervision of rehabilitation specialists. Cognitive stimulation techniques and occupational therapy strategies were integrated to support activities of daily living (ADL).

After discharge, patients continued individualized exercise programs, combining progressive walking, aerobic training, and balance re-education to enhance independence and quality of life. The post-COVID-19 rehabilitation program was initiated immediately after clinical stabilization and aimed to restore functional capacity through a progressive, stage-based approach.

The intervention combined respiratory retraining, muscle strengthening, postural control, and aerobic reconditioning, adapted to each patient’s functional level (Stages 1–5). Sessions were supervised by physical therapists and lasted 30–45 min, five times per week during hospitalization, followed by 10 outpatient sessions every three months for up to one year.

The structured rehabilitation protocol was organized in five stages, from bed-level mobilization to advanced dynamic training, as summarized below:

In-hospital rehabilitation (Stages 1–3)

Patients were hospitalized for 10 days at the Dr. Carol Davila Central Military Emergency University Hospital during the acute phase of SARS-CoV-2 infection. After antiviral therapy and stabilization, they entered the rehabilitation program upon eligibility and informed consent.

- Stage 1: Early mobilization and diaphragmatic breathing in supine, lateral, and prone positions every 2 h; spirometer training (3×/day); simple upper and lower limb exercises (10 repetitions, 3 sets/day).
- Stage 2: Transition to sitting and gradual verticalization, maintaining upright posture for 10–120 s under supervision; continued respiratory training.
- Stage 3: Independent standing and short-distance walking (3–10 m), 3 repetitions every 3 h, supervised by physiotherapists.

Outpatient rehabilitation (Stages 4–5)

After discharge, patients resumed therapy within 30 days, using the Huber 360 Evolution platform (LPG^®^ Systems, Valence, France) for dynamic postural and balance recovery.

- Stage 4: Postural control and balance re-education (2 sessions/week for 10 days), progressing from level 1 to level 5 based on hemodynamic stability.
- Stage 5: Dynamic Fortification Program—targeted strengthening of hip, knee, and ankle stabilizers (2 sessions/week, 10 repetitions per session).

Across all stages, the program emphasized breathing control, progressive effort tolerance, and patient education to prevent deconditioning and promote independence.

All rehabilitation sessions were conducted under the supervision of physical therapists, following an initial assessment and medical supervision provided by a physiatrist.

Table 1 presents the structured rehabilitation protocol implemented in post-COVID-19 patients, detailing the main objectives, exercises, frequency, and progression criteria for each rehabilitation phase.

### 2.3. Functional Assessments

**10-Meter Walk Test (10MWT):**

Walking speed was assessed over a 10 m central segment of a 14 m walkway, with 2 m acceleration and deceleration zones [46]. Tests were performed at both comfortable and maximum safe speed. Walking speed was calculated as distance/time (m/s). For interpretation, commonly used cutoffs were applied [47]:

- <0.4 m/s: very limited walking capacity, mainly indoors;
- 0.4–0.8 m/s: limited community ambulation;
- 0.8–1.2 m/s: functional community ambulation;
- 1.2 m/s: normal walking capacity and independence.

**Timed Up and Go (TUG):**

The TUG test measured the time (in seconds) required to rise from a chair, walk 3 m, turn, return, and sit down. Mobility categories were defined as:

- ≤10 s: normal mobility;
- 10–20 s: good mobility, independent community ambulation;
- 20–30 s: mobility impairment, requiring an assistive device;
- 30 s: severely impaired mobility [26,48,49].

Age-specific normative values were considered for interpretation, as previously reported in the literature [50,51].

Both assessments were performed at hospital admission (baseline), at discharge after completion of the initial rehabilitation program, and at 3-month intervals following discharge, up to 12 months post-discharge.

For interpretation, TUG values were classified into: Category A (≤10 s, normal mobility), Category B (10–20 s, good mobility), and Category C (20–30 s, impaired mobility). Similarly, the 10MWT was categorized as: <0.4 m/s (very limited functional walking), 0.4–0.8 m/s (limited community ambulation), 0.8–1.2 m/s (functional community ambulation), and D > 1.2 m/s (normal walking, independent).

To facilitate dichotomous comparisons, TUG categories A and B were grouped as “functional”, and C as “non-functional”, while for 10MWT, categories B and C (and D, where applicable) were grouped as “functional” and A as “non-functional”.

Statistical analyses focused on the evolution between the two key timepoints—baseline and 12 months post-discharge—and on the associations between the TUG and 10MWT outcomes.

### 2.4. Statistical Analysis

Data were analyzed using IBM SPSS Statistics version 25 (IBM Corp., Armonk, NY, USA) and Microsoft Excel 2024.

Quantitative variables were expressed as the mean ± standard deviation (SD) or median (interquartile range, IQR), according to the data distribution assessed with the Shapiro–Wilk test. Categorical variables were expressed as frequencies and percentages.

Between-group comparisons were conducted using independent *t*-tests or Mann–Whitney U tests, as appropriate. Paired data were analyzed with paired *t*-tests or Wilcoxon signed-rank tests. For comparisons across more than two groups, ANOVA or Kruskal–Wallis tests were applied, followed by post hoc Bonferroni-corrected analyses. Correlations were assessed using Pearson’s or Spearman’s coefficients, depending on variable distribution.

For categorical data, Chi-square or Fisher’s exact tests were used for group comparisons, with Bonferroni correction applied to pairwise analyses.

Within-group comparisons of dichotomized outcomes (functional vs. non-functional) were performed using the McNemar test for paired data, while Pearson’s Chi-square test was used to evaluate between-group differences and associations between categorical variables (e.g., TUG and 10MWT classifications).

Effect sizes for categorical associations were expressed as Cramér’s V, and for paired binary data as

$$r=\frac{Z}{\sqrt{N}}$$

where Z = √(χ^2^). Values of r = 0.10, 0.30, and 0.50 were interpreted as small, moderate, and large effects, respectively.

For ordinal comparisons between the initial and final categories (A, B, C), non-parametric tests for related samples were used where appropriate.

All tests were two-tailed, and a *p*-value < 0.05 was considered statistically significant. Effect sizes and 95% confidence intervals (CI) were calculated where applicable.

Statistical analyses were designed to evaluate both within-subject improvements following rehabilitation and between-group differences, as well as explore the associations between TUG and 10MWT outcomes as complementary indicators of functional recovery.

## 3. Results

### 3.1. Baseline Characteristics

A total of 193 patients with mild to moderate post-COVID-19 conditions were included in the study, of whom 115 (59.59%) were women and 78 (40.41%) were men. The mean age was 59.79 ± 13.21 years (range: 26–91). At baseline, most patients presented with mobility limitations, reflected in prolonged TUG times and reduced gait speed at the 10MWT. Older adults (≥60 years) presented with significantly lower baseline functional scores compared to younger participants (<60 years). Detailed demographic data, stratified by sex and age group (<60 vs. ≥60 years), are provided in Table 2.

### 3.2. Evolution of TUG Performance

At baseline, most patients in the study group (45%) were classified as mobility impaired (category C), while only 30.6% had normal mobility (category A). After rehabilitation, 60.6% reached normal mobility and 32.5% good mobility, with only 6.9% remaining impaired. These improvements were statistically significant (*p* < 0.001).

Within-group comparisons indicated significant transitions from impaired to normal mobility (categories C and A, *p* < 0.001), while intermediate changes (category B) were not significant (*p* = 0.154).

In contrast, the control group showed no meaningful improvement, with most participants remaining in category C. Between-group analysis confirmed that the improvement rate was significantly higher in the study group (*p* < 0.001).

Detailed distributions are shown in Table 3.

### 3.3. Evolution of Gait Speed at the 10MWT

At baseline, most patients in the study group were classified in categories A (48.1%) and B (50.6%), with only 1.3% achieving community-level functional ambulation (C). After one year of rehabilitation, the distribution shifted significantly toward higher functional categories: 14.4% remained in A, 60% in B, and 25.6% reached category C (*p* < 0.001). The within-group McNemar analysis confirmed significant improvement for categories A and C (*p* < 0.001 for both), indicating a substantial enhancement of walking ability and endurance following the rehabilitation program.

In contrast, the control group showed limited changes: most participants remained in categories A–B, with only a minor increase in category C (from 0% to 9.1%). Between-group comparison demonstrated a significant difference in functional evolution (*p* < 0.001), supporting the positive impact of the structured rehabilitation program on gait performance and the recovery of community ambulation capacity. Detailed distributions are shown in Table 4.

### 3.4. Functional Mobility and Walking Speed (TUG and 10MWT)

When analyzed as a binary outcome (functional vs. non-functional), the proportion of patients classified as functionally independent on the TUG test increased after rehabilitation, although the change did not reach statistical significance (McNemar χ^2^ = 2.44, *p* = 0.118, r = 0.12), indicating a small effect size.

This trend, however, was consistent with the categorical (A/B/C) analysis, which demonstrated a significant overall shift toward improved mobility levels following rehabilitation (Cramér’s V = 0.468, *p* < 0.001, N = 187), corresponding to a moderate-to-large effect.

For the 10-Meter Walk Test (10MWT), within-group analysis demonstrated a significant improvement in walking capacity after rehabilitation (McNemar χ^2^ = 10.30, *p* = 0.001, r = 0.24), reflecting a moderate effect size.

The proportion of patients achieving functional walking speeds increased substantially from baseline to follow-up, in line with the categorical analysis that showed a large between-group difference (Cramér’s V = 0.500, *p* < 0.001, N = 187).

A strong and statistically significant association was also found between the TUG and 10MWT functional classifications at follow-up (χ^2^ = 29.44, *p* < 0.001, Cramér’s V = 0.40), corresponding to a moderate-to-strong effect.

Patients categorized as functionally independent according to the TUG were also more likely to demonstrate normal or community-level gait speeds on the 10MWT.

This concordance underscores the internal consistency and convergent validity of the two mobility assessments in quantifying rehabilitation outcomes.

## 4. Discussion

### 4.1. Overview of Main Findings

The present controlled study demonstrates that patients with mild to moderate post-COVID-19 conditions achieved significant improvements in both functional mobility and gait speed following a structured rehabilitation program, as assessed by the TUG and 10MWT tests. Compared with the control group, participants in the rehabilitation cohort showed a marked redistribution toward higher functional categories, with a moderate-to-large effect size for TUG (Cramér’s V = 0.468, *p* < 0.001) and a large effect for 10MWT (Cramér’s V = 0.500, *p* < 0.001). Within-group comparisons further supported these findings: while the improvement in TUG functional classification did not reach statistical significance (McNemar χ^2^ = 2.44, *p* = 0.118, small effect), a significant change was observed for 10MWT (McNemar χ^2^ = 10.30, *p* = 0.001, moderate effect). These results indicate that rehabilitation produced measurable gains in gait performance and mobility consistency across complementary functional assessments.

Our results align with previous reports showing persistent deficits in mobility and exercise capacity among COVID-19 survivors [38,39]. Importantly, while most published studies have focused on patients with severe disease or those requiring intensive care, our cohort highlights that functional impairments are also common in mild-to-moderate cases. This underlines the clinical importance of early rehabilitation, consistent with the growing recognition that COVID-19 sequelae extend beyond the acute phase and across all severity levels [36,37].

### 4.2. Role of Functional Tests (TUG and 10MWT) in Post-COVID Assessment

Walking speed and TUG scores declined after 2020 and continued to worsen afterward. Therefore, the impact of lifestyle changes related to the pandemic on community-dwelling individuals with disabilities was not temporary [49]. Functional mobility tests such as TUG and 10MWT provide rapid, reliable, and low-cost methods for assessing motor performance and identifying individuals at increased risk of mobility impairment. The TUG test, originally validated in elderly populations [23], and subsequently applied to stroke, frailty, and neurological disorders [30,52], has also been proposed as a screening tool for fall risk in older adults, with longer completion times being associated with a higher probability of falls [26,27,28]. When interpreted alongside complementary clinical measures, TUG contributes significantly to the multidimensional evaluation of balance and fall risk. The 10MWT, on the other hand, isolates gait speed—a parameter strongly associated with independence and mortality risk [29]. In the context of COVID-19, most studies have favored the Six-Minute Walk Test (6MWT) as an outcome measure [36,38]. However, our results show that the 10MWT is equally capable of detecting clinically relevant improvements in walking capacity after rehabilitation. In our cohort, post-rehabilitation functional classifications based on the TUG and 10MWT were strongly correlated (χ^2^ = 29.44, *p* < 0.001, Cramér’s V = 0.40), confirming the internal consistency and concurrent validity of these two measures in capturing mobility recovery.

Short-distance walk tests such as the 10MWT offer practical advantages in settings where longer corridors are unavailable, making them especially suited for outpatient programs or constrained environments [31].

A previous study highlighted the value of the 6MWT in documenting post-COVID recovery trajectories [42], demonstrating its strong sensitivity in detecting both respiratory and functional impairments. When combined, TUG, 10MWT, and 6MWT provide complementary perspectives on recovery, spanning balance, speed, and endurance. This multidimensional approach enhances the robustness of functional assessment and should be considered a gold standard in rehabilitation practice [19,34].

### 4.3. Improvements After Rehabilitation

Our study revealed significant improvements in both TUG and 10MWT outcomes following the structured rehabilitation program compared with the control group. For TUG, the redistribution of patients from the impaired (category C) to normal/good performance (categories A and B) illustrates gains in balance, transitions, and functional independence. Although the binary within-group change (functional vs. non-functional) did not reach statistical significance (McNemar χ^2^ = 2.44, *p* = 0.118, r = 0.11, small effect), the categorical analysis confirmed a significant overall shift toward higher functional levels (Cramér’s V = 0.468, *p* < 0.001). Similarly, the 10MWT showed a significant improvement in walking speed categories (McNemar χ^2^ = 10.30, *p* = 0.001, r = 0.24, moderate effect), with a reduction in patients exhibiting severely limited ambulation (<0.4 m/s) and an increase in those achieving functional community walking (0.8–1.2 m/s). These findings highlight both statistical and clinical relevance, confirming the impact of rehabilitation on mobility restoration.

These findings are consistent with evidence showing that structured rehabilitation improves walking ability, postural control, and overall independence in post-COVID populations [33,35]. In a complementary study, significant reductions in perceived effort and breathlessness were observed following a standardized post-COVID rehabilitation program, supporting the multidimensional benefits of such interventions [53]. Beyond functional outcomes, aerobic and resistance training interventions have been shown to reduce fatigue, enhance psychological well-being, and improve cardiovascular parameters [12,40].

Structured rehabilitation has consistently been proven beneficial across a wide spectrum of medical conditions, reinforcing its universal role in restoring functional capacity and quality of life. Evidence from orthopedic [54,55,56,57], neurological [58,59,60], and cardiopulmonary rehabilitation programs [61,62,63,64,65,66] demonstrates that individualized, multidisciplinary approaches lead to significant improvements in mobility, endurance, and autonomy. These findings support the broader applicability of structured rehabilitation principles observed in our cohort, confirming that similar mechanisms of neuromuscular adaptation and functional reconditioning underpin recovery across diverse clinical contexts.

### 4.4. Age and Sex Differences in Recovery

One notable finding was the robust improvement among older adults (≥60 years), who achieved functional gains comparable to, or greater than, younger patients. This challenges the assumption that advanced age limits rehabilitation potential. Our results echo findings from [34,67], which highlight that older adults may experience larger relative improvements because of lower baseline performance. Moreover, age-related deficits such as sarcopenia and balance impairment appear responsive to targeted training, particularly when interventions combine mobility, strength, and respiratory exercises [68,69,70,71].

Researchers have shown that in adults aged 65 years and older who participated in an eight-week exercise program performed twice weekly, both the TUG and 10MWT scores improved significantly, confirming that functional performance in this age group remains highly responsive to structured rehabilitation [72].

The TUG performance showed that as age increases, hospitalization duration and comorbidity burden tend to rise, negatively affecting mobility and balance. This is reflected in the longer time required to complete the test [73]. Most published studies have applied TUG and 10MWT primarily in patients with severe post-COVID-19 disease, whereas data on mild and moderate forms remain limited.

Ampt et al. examined TUG performance in older adults recovering from mild COVID-19 and reported significantly prolonged completion times compared with healthy controls [33]. Similarly, Kowal et al. [48] found that individuals with previous COVID-19 infection exhibited persistent alterations in joint range of motion and increased TUG duration even eight weeks post-discharge, depending on disease severity.

In patients with severe infection, Sirayder et al. [74] reported significantly poorer TUG scores compared with controls, indicating sustained deficits in functional capacity. Joaquín et al. [75] also observed a decline exceeding 40% in TUG performance among post-ICU COVID-19 patients, underlining the long-term impact of critical illness.

Curci et al. [76] further demonstrated that post-acute COVID-19 patients enrolled in a structured inpatient rehabilitation program (30 min/session, twice daily) achieved statistically significant gains in the 6-Minute Walk Test (6MWT), confirming the responsiveness of functional capacity to rehabilitation interventions even after severe disease.

Sex-related differences were also observed, with both men and women showing significant functional recovery, though men demonstrated a more uniform elimination of severely impaired mobility (TUG-C). These results partially mirror reports from international studies, which have suggested sex-based differences in post-COVID fatigue and physical performance [19]. However, our data suggest that both sexes benefit substantially from rehabilitation, indicating that sex should not be a limiting factor in the prescription of such programs.

### 4.5. Complementary Value of TUG and 10MWT

While both tests measure aspects of functional mobility, their differences justify their combined use. The TUG test integrates complex functional tasks—sit-to-stand transitions, turning, and walking—providing insight into balance and fall risk [25,26]. The 10MWT isolates gait speed, a well-established predictor of community participation and survival [31].

Our correlation analysis demonstrated a strong and significant association between the two measures (χ^2^ = 29.44, *p* < 0.001, Cramér’s V = 0.40), supporting their concurrent validity. However, discrepancies were noted among patients with greater baseline impairments: some individuals categorized as severely limited on the TUG displayed only moderate deficits on the 10MWT, and vice versa. This reflects the fact that gait speed alone may underestimate impairments in balance and transitional mobility. Previous research confirms that the TUG and 10MWT should be interpreted complementarily to avoid overlooking critical aspects of function [24,32].

### 4.6. Comparison with Existing Evidence

A growing body of evidence supports the role of rehabilitation in mitigating post-COVID sequelae. Longitudinal studies have documented persistent impairments in endurance, muscle strength, and mobility months after infection [37,77]. Pulmonary rehabilitation programs have demonstrated benefits for severe patients [38], but recent findings emphasize the need for structured rehabilitation even in mild-to-moderate populations [33].

Our findings are consistent with previous reports showing improvements in mobility tests after rehabilitation in post-COVID patients [35,39]. Importantly, by including a control group and quantifying effect sizes, the present study extends existing evidence by confirming that measurable functional recovery also occurs in mild-to-moderate cases, which remain underrepresented despite comprising the majority of infections.

Our results also align with evidence from broader rehabilitation contexts. For instance, extracorporeal shockwave therapy has demonstrated benefits not only in musculoskeletal conditions [78,79,80,81,82], but also in supporting respiratory recovery in patients affected by coronavirus disease 2019 (COVID-19), where its anti-inflammatory and circulatory effects have been shown to enhance breathing efficiency and chest wall mobility [83]. Similarly, structured rehabilitation interventions—including robot-assisted therapy, functional electrical stimulation, and aerobic reconditioning—have proven effective in neurological and cardiopulmonary disorders, emphasizing the cross-cutting benefits of individualized, multidisciplinary programs across diverse clinical populations. These diverse findings highlight that structured rehabilitation—whether exercise-based or device-assisted—consistently improves mobility, function, and quality of life.

Moreover, recent studies confirm the persistence of post-COVID musculoskeletal, respiratory, and neurological impairments, reinforcing the urgent need for targeted interventions [48,84,85,86,87]. Our results extend this evidence by demonstrating that standardized functional tests capture meaningful recovery in everyday clinical practice.

### 4.7. Clinical and Public Health Implications

Our controlled results demonstrate that rehabilitation significantly enhances mobility and gait recovery even in mild-to-moderate post-COVID cases, supporting the expansion of such programs beyond severe disease survivors. Individuals recovering from non-severe infections frequently exhibit functional deficits that affect daily autonomy and quality of life, reinforcing the need for early, structured intervention. Given the high prevalence of these cases, rehabilitation should be systematically integrated into both hospital-based and community-level healthcare pathways [18,88,89,90]. Similar disruptions and adaptations have been reported in other chronic conditions, including cardiac rehabilitation for patients with diabetes and cardiovascular comorbidities, where the pandemic accelerated the adoption of structured, multidisciplinary approaches to maintain care continuity [91]. Beyond traditional in-person programs, tele-rehabilitation approaches initially developed for chronic respiratory diseases [92,93] could be adapted to support post-COVID patients, ensuring continuity of care and broader accessibility.

Furthermore, promoting physical activity as a preventive and restorative measure is essential. Sedentarism, exacerbated by quarantine and social restrictions, remains a major risk factor for adverse outcomes [16,94]. Public health initiatives should therefore encourage structured activity at both the individual and population levels, particularly in vulnerable groups such as older adults and those with comorbidities.

### 4.8. Implications for Service Design

Beyond individual gains, the present findings strongly support the integration of structured rehabilitation programs throughout the continuum of post-COVID care, encompassing early referral pathways, standardized functional screening using the TUG and 10MWT at both admission and discharge, and scalable delivery models—including in-clinic, community, and home-based interventions supported by tele-monitoring. Technology-assisted platforms have already demonstrated significant benefits for improving motor control, balance, and coordination in chronic rehabilitation settings [95]. Embedding brief, progressive aerobic and balance-oriented exercise, coupled with education on activity pacing and fall prevention, may optimize outcomes while using limited resources efficiently. Importantly, service models should prioritize older adults and those with comorbidities—groups that, in our cohort, demonstrated substantial capacity for improvement—while ensuring longitudinal follow-up to maintain functional gains, promote independence, and sustain community participation [96,97,98,99,100].

### 4.9. Limitations and Future Directions

This study has several limitations. Although a control group was included to strengthen the comparative analysis, the retrospective observational design still limits causal inference. Additionally, functional outcomes were limited to the TUG and 10MWT, without complementary objective measures such as cardiopulmonary testing or muscle strength assessments.

Follow-up assessments were limited to the one-year post-rehabilitation period, which, while sufficient to capture short- and medium-term outcomes, did not allow for an evaluation of the long-term sustainability of functional gains. Another limitation is the absence of the Six-Minute Walk Test (6MWT), widely recognized as a reliable measure of endurance and overall functional capacity in post-COVID-19 populations. Although the TUG and 10MWT provide valuable information regarding balance, mobility, and gait speed, the addition of the 6MWT could have complemented these measures by capturing endurance and cardiorespiratory recovery.

Future research should build upon these findings through prospective, multicenter studies using standardized rehabilitation protocols and multidimensional outcome measures, including psychological and quality-of-life assessments, biomarkers of recovery, and advanced gait analysis. Such approaches would help establish causal relationships and provide a more comprehensive understanding of post-COVID rehabilitation trajectories.

## 5. Conclusions

This study provides comparative evidence that structured rehabilitation is effective in improving functional mobility and gait capacity in patients recovering from mild to moderate COVID-19. Using two complementary assessments—Timed Up and Go (TUG) and the 10-Meter Walk Test (10MWT)—we demonstrated significant gains in mobility, balance, and walking speed across the entire cohort, confirming the clinical value of targeted rehabilitation interventions.

Functional mobility (TUG). The most notable improvement was observed in the reduction in patients classified in the poorest mobility category, accompanied by a marked increase in those reaching normal or near-normal functional levels. The between-group comparison showed a moderate-to-large effect size (Cramér’s V = 0.468, *p* < 0.001), while the within-group analysis revealed a consistent trend toward functional improvement. These results highlight the capacity of rehabilitation to reverse mobility impairments, even in patients who initially present with substantial deficits in dynamic balance, transitional movements, and stability.

Gait speed (10MWT). Rehabilitation also led to significant enhancements in walking capacity. The redistribution of patients from very low gait speeds (<0.4 m/s) toward functional community ambulation (0.8–1.2 m/s) underscores the potential for meaningful recovery in locomotor function. The observed effect was large (Cramér’s V = 0.500, *p* < 0.001), and the McNemar test confirmed a moderate within-group change (r = 0.24, *p* = 0.001). Importantly, the shift toward slower but safer walking patterns in certain subgroups should not be interpreted as regression, but rather as a protective adaptation that promotes independence and reduces fall risk.

Influence of age and sex. Both younger and older patients responded favorably to the program. Contrary to common assumptions, older adults (≥60 years) not only improved but in some instances demonstrated greater relative gains than younger patients, suggesting that age is not a limiting factor for rehabilitation success. Similarly, outcomes were comparable between women and men, reinforcing the broad applicability of rehabilitation interventions across demographic subgroups.

Complementary nature of TUG and 10MWT. Although both tests improved significantly, they capture distinct facets of motor performance. TUG is more sensitive to balance and transitional tasks, while 10MWT isolates walking speed. Their strong association (Cramér’s V = 0.40, *p* < 0.001) supports their combined use for a comprehensive evaluation of recovery trajectories.

Taken together, these findings challenge the assumption that only patients with severe COVID-19 require rehabilitation. Functional limitations remain prevalent even in mild and moderate cases, but they are responsive to intervention. By integrating structured, evidence-based rehabilitation protocols into the continuum of COVID-19 care, clinicians can promote meaningful improvements in independence, community mobility, and quality of life for a broad spectrum of patients.

## Figures and Tables

**Table 1:** Structure of the post-COVID-19 Rehabilitation Program.

Phase/Setting	Main Objectives	Exercises and Techniques	Frequency/Duration	Progression Criteria
Early phase (bed-based rehabilitation)	Prevent deconditioning, improve respiratory efficiency	- Diaphragmatic and abdominal breathing (supine, knees flexed) - Postural drainage and bronchial clearance - Position changes every 2 h (supine, lateral, prone)	5 sessions/day × 10 days	Stable oxygen saturation, tolerance to sitting
Mobilization and flexibility training	Facilitate trunk control and limb mobility	- Upper limb flexion/extension, abduction (10 reps × 5/day) - Lower limb flexion/extension, dorsiflexion, plantar flexion (10 reps × 5/day) - Supported sitting at bedside	Daily during hospitalization	Independent sitting and tolerance to orthostatism
Verticalization and gait initiation	Improve balance and prevent orthostatic hypotension	- Assisted standing and gait initiation under supervision	Daily during late inpatient phase	Independent gait initiation
Outpatient rehabilitation (Huber 360 Evolution)	Enhance strength, coordination, posture, and respiratory capacity	- Multi-axial dynamic balance training - Hip/knee/ankle stabilizer strengthening - Cognitive and aerobic exercises for endurance	2 sessions/week × 30 min, 10 sessions per cycle (every 3 months, up to 1 year)	Achievement of functional level 4–5
Final goals	- Increase flexibility and muscle strength - Improve axial stability and coordination - Restore independence in ADLs - Enhance quality of life	—	—	—

Note: The rehabilitation protocol was standardized for all patients according to their functional capacity at admission, with gradual adaptation to tolerance and performance. Outpatient sessions were conducted in the hospital’s rehabilitation department under the supervision of the physical therapists.

**Table 2:** Baseline characteristics of the study cohort.

Characteristic	Total (n = 193)	Study Group (n = 160)	Control Group (n = 33)
Age (years), mean ± SD	58.79 ± 13.21	58.54 ± 13.30	59.97 ± 12.92
Age group, n (%)			
<60 years	48.11 ± 7.23	47.98 ± 7.33	48.81 ± 6.87
≥60 years	70.64 ± 6.51	70.68 ± 6.47	70.47 ± 6.92
Sex, n (%)			
Women	115 (59.59%)	97 (60.63%)	18 (54.54%)
Men	78 (40.41%)	63 (39.37%)	15 (45.46%)

**Table 3:** Distribution of TUG categories before and after the rehabilitation program by study and control groups.

Group		n	Pre A (n, %)	Pre B (n, %)	Pre C (n, %)	Post A (n, %)	Post B (n, %)	Post C (n, %)	*p*-Value
Study group		160	49 (30.63%)	39 (24.37%)	72 (45.00%)	97 (60.63%)	52 (32.50%)	11 (6.88%)	<0.001
	Women	97	31 (31.96%)	23 (23.71%)	43 (44.33%)	60 (61.86%)	26 (26.80%)	11 (11.34%)	
	Men	63	18 (28.57%)	16 (25.40%)	29 (46.03%)	37 (58.73%)	26 (41.27%)	0	
	<60 years	86	37 (43.53%)	23 (27.06%)	25 (29.41%)	64 (75.29%)	17 (20.00%)	4 (4.71%)	
	≥60 years	74	12 (16.22%)	16 (21.62%)	46 (62.16%)	33 (44.59%)	34 (45.95%)	7 (9.46%)	
Control group		33	0	25 (75.76%)	8 (24.24%)	4 (12.12%)	8 (24.24%)	21 (63.64%)	0.125
	Women	18	0	13 (72.22%)	5 (27.78%)	1 (5.56%)	5 (27.77%)	12 (66.67%)	
	Men	15	0	12 (80.00%)	3 (20.00%)	3 (20.00%)	3 (20.00%)	9 (60.00%)	
	<60 years	16	0	15 (93.75%)	1 (6.25%)	3 (18.75%)	8 (50.00%)	5 (31.25%)	
	≥60 years	17	0	10 (58.82%)	7 (41.18%)	1 (5.88%)	0	16 (94.12%)	

Note: Within-group comparisons were assessed using the McNemar test for paired categorical data (functional vs. non-functional). Abbreviations: A—normal mobility (≤10 s); B—good mobility (10–20 s); C—mobility impairment (≥20 s). For statistical analysis, categories A and B were grouped as functional, while category C was considered non-functional. Values are expressed as counts and percentages. Statistical significance was set at *p* < 0.05.

**Table 4:** Evolution of gait speed at the 10MWT.

Group		n	Pre A (n, %)	Pre B (n, %)	Pre C (n, %)	Post A (n, %)	Post B (n, %)	Post C (n, %)	*p*-Value
Study group		160	77 (48.13%)	81 (50.63%)	2 (1.25%)	23 (14.38%)	96 (60.00%)	41 (25.63%)	< 0.001
	Women	97	45 (46.39%)	51 (52.58%)	1 (1.03%)	18 (18.56%)	55 (56.70%)	24 (24.74%)	
	Men	63	32 (50.79%)	30 (47.62%)	1 (1.59%)	5 (7.94%)	41 (65.08%)	17 (26.98%)	
	<60 years	86	26 (30.23%)	58 (67.44%)	1 (1.16%)	9 (10.47%)	41 (47.67%)	35 (40.70%)	
	≥60 years	74	50 (67.57%)	23 (31.08%)	1 (1.35%)	14 (18.92%)	54 (72.97%)	6 (8.11%)	
Control group		33	5 (15.15%)	28 (84.85%)	0	13 (39.39%)	17 (51.52%)	3 (9.09%)	<0.001
	Women	18	2 (11.11%)	16 (88.89%)	0	7 (38.89%)	8 (44.44%)	3 (16.67%)	
	Men	15	3 (20.00%)	12 (80.00%)	0	6 (40.00%)	9 (60.00%)	0	
	<60 years	16	0	16 (100%)	0	1 (6.25%)	12 (75.00%)	3 (18.75%)	
	≥60 years	17	5 (29.41%)	12 (70.59%)	0	12 (70.59%)	5 (29.41%)	0	

## Data Availability

The data presented in this study are available on request from the corresponding author. The data are not publicly available due to privacy and ethical restrictions.
